# Supplementary figures and images for: Differential carbonic anhydrase activities control EBV-induced B-cell transformation and lytic cycle reactivation
Source: PLoS Pathog. 2024 Mar 26;20(3):e1011998. doi: 10.1371/journal.ppat.1011998 (PMC10997083; doi:10.1371/journal.ppat.1011998)

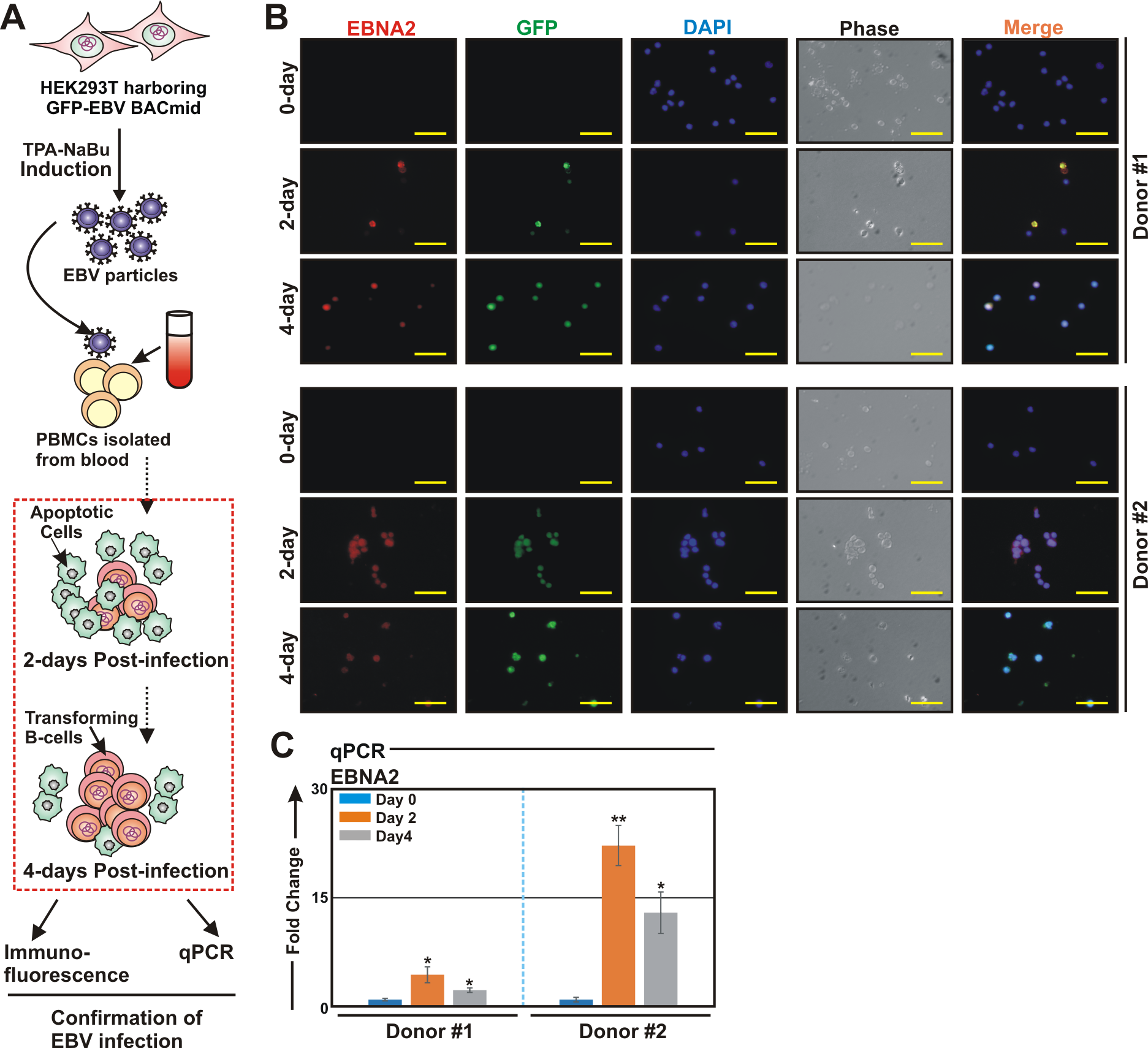

Supplement: S1 Fig — (A) Schematic representation of EBV infection (MOI ~10) in primary B-lymphocytes (PBMC) for 2 and 4 days. (B) Immunofluorescence study showing EBV nuclear antigen EBNA2 and GFP expressions. (C) Relative changes of EBNA2 expression in response to EBV infection of primary B-lymphocytes are represented as bar diagrams in comparison to uninfected control samples using B2M as housekeeping gene. Error bars represent standard deviations of triplicate assays of two independent experiments. *, **, *** = p-value < 0.01, 0.005 and 0.001 respectively. (TIF) [file ppat.1011998.s001.TIF]

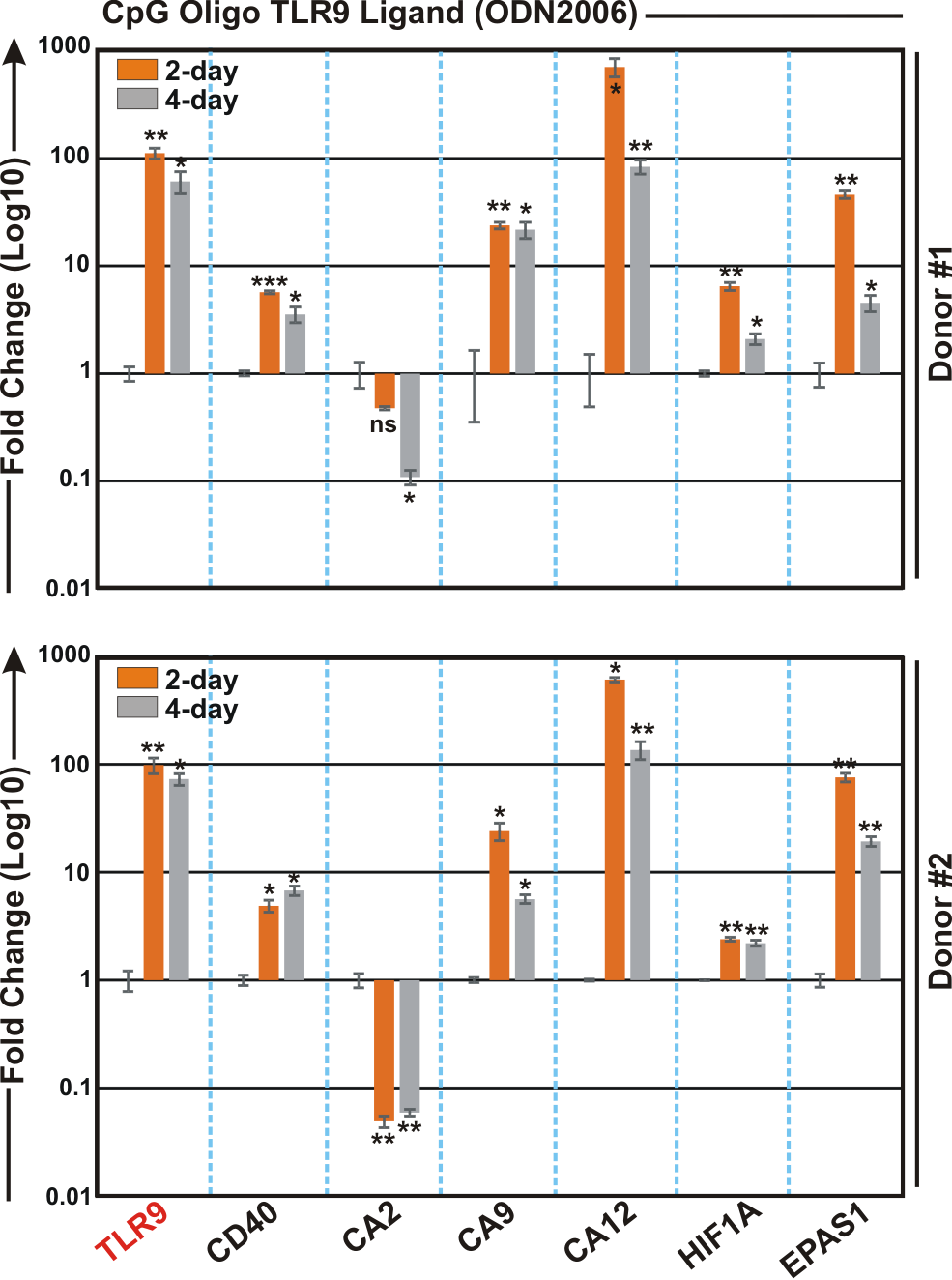

Supplement: S2 Fig — ~10 x 106 PBMCs isolated from two individual donors were treated with CpG oligo TLR9 ligand ODN2006 for the indicated time points. 2-days and 4-days post-treated cells were harvested, isolated total RNA and subjected for qPCR analyses after converting them to cDNA. The relative changes in transcripts (log10) of the selected genes using the 2-ΔΔCt method are represented as bar diagrams in comparison to untreated PBMC-control using B2M as housekeeping gene. Two independent experiments were carried out in similar settings and results represent as an average value for each transcript. *, **, *** = p-value < 0.01, 0.005 and 0.001 respectively. (TIF) [file ppat.1011998.s002.TIF]

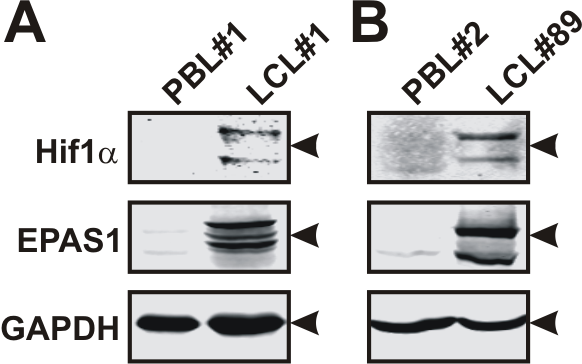

Supplement: S3 Fig — ~5 x 10^6^ PBMCs isolated from two individual donors and two LCL clones (LCL#1 and LCL#89) were harvested, washed with 1 x PBS, lysed in RIPA buffer and subjected for western blot analyses with the indicated antibodies. GAPDH blot was used as loading control. Representative gel pictures are shown of at least two independent experiments. (TIF) [file ppat.1011998.s003.TIF]

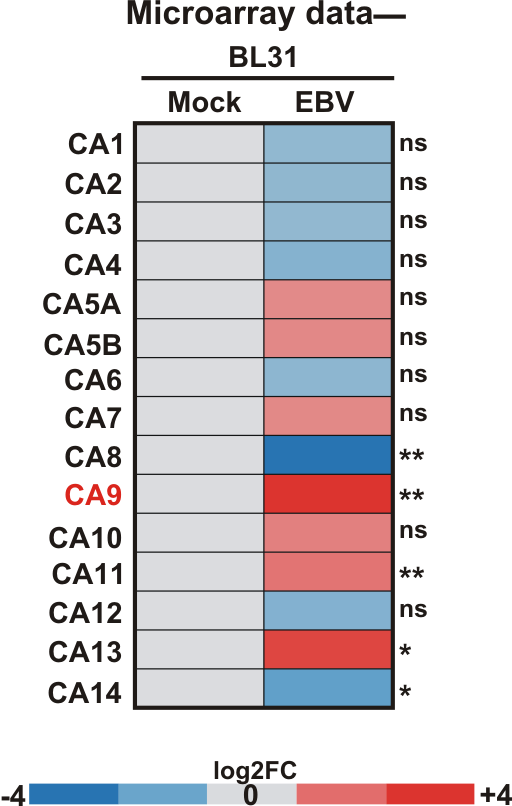

Supplement: S4 Fig — Heat map representation of the selected genes of microarray data [36] of EBV infected BL31 cell line. (TIF) [file ppat.1011998.s004.TIF]

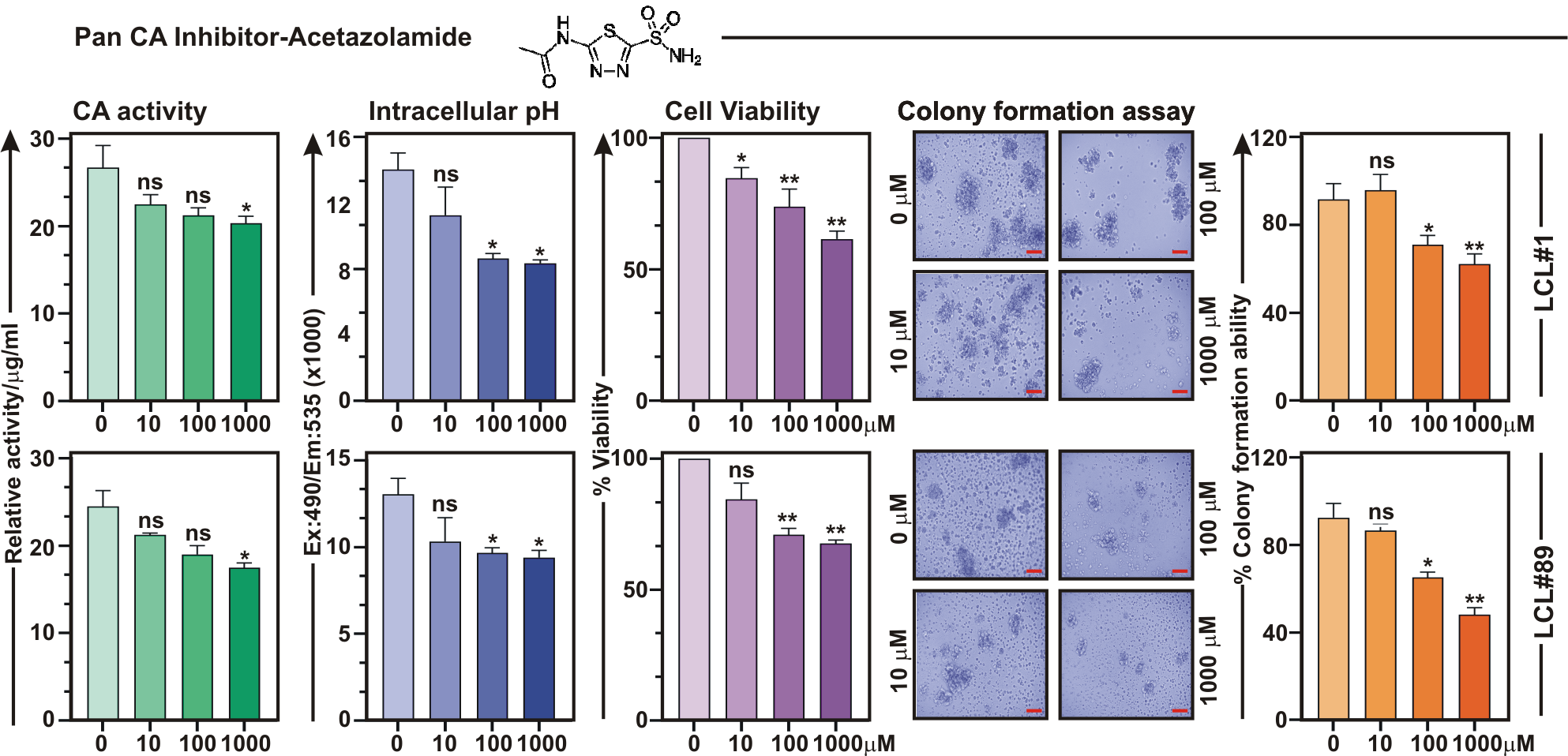

Supplement: S5 Fig — LCLs (LCL#1 and LCL#89) were either left untreated (DMSO control) or treated with increasing concentrations (10–1000 μM) of pan carbonic anhydrase inhibitor acetazolamide for 24 h and measured carbonic anhydrase activity, intracellular pH, cell viability and colony formation ability as described in the “Materials and Methods section”. Carbonic anhydrase activity and intracellular pH determination assays were performed using kits as per manufacturer’s protocols. For cell viability assays, viable cells from each well of 6-well plates were measured by Trypan blue exclusion method using an automated cell counter. For the colony formation assay, 14 days post-treatment colonies on the soft agar were photographed (bright-field) using a Fluorescent Cell Imager. Scale bars, 100 μm. The number of colonies were measured by ImageJ2 software and plotted as bar diagrams. Error bars represent standard deviations of duplicate assays of two independent experiments. *, **, *** = p-value < 0.01, 0.005 and 0.001 respectively. (TIF) [file ppat.1011998.s005.TIF]

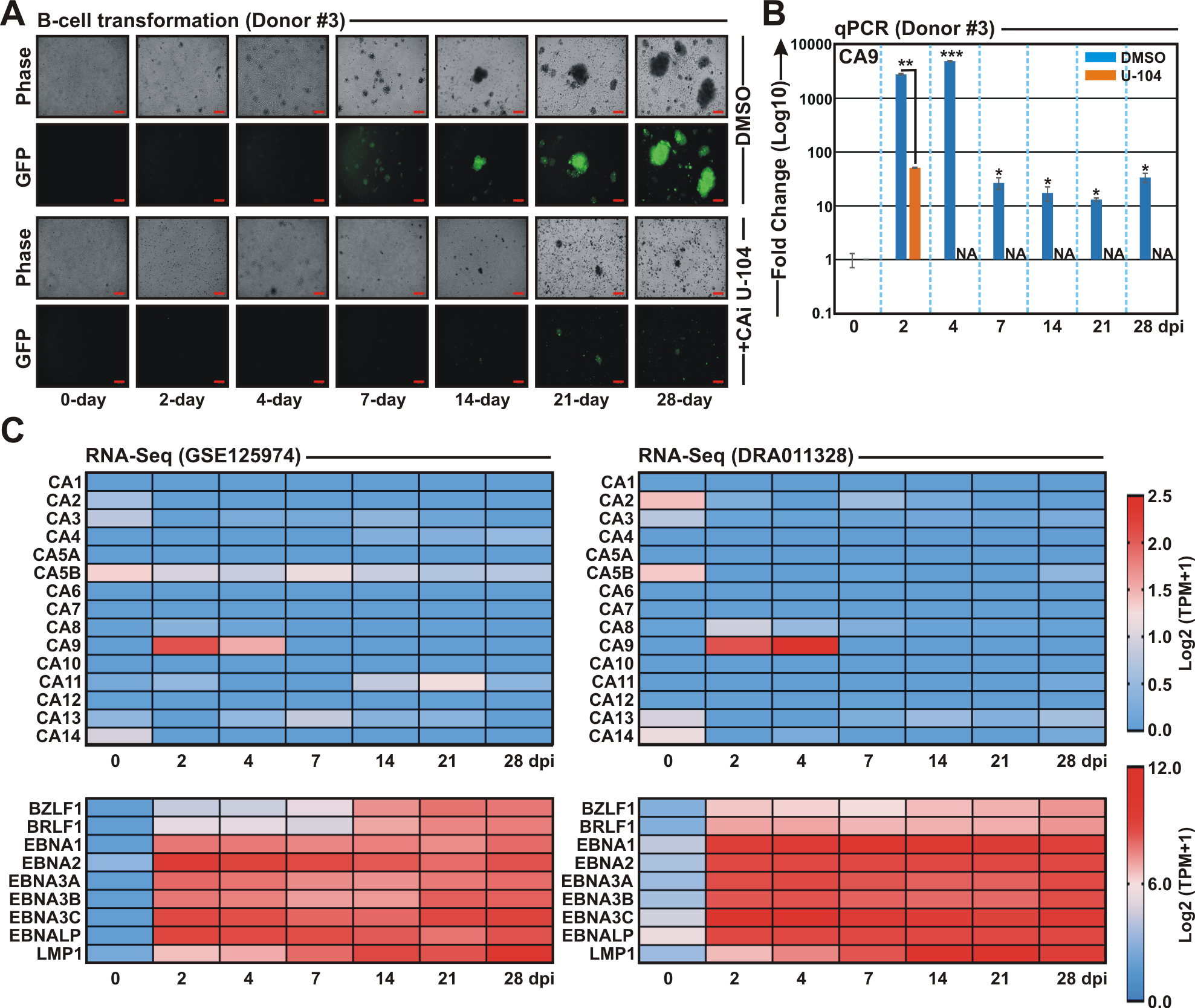

Supplement: S6 Fig — (A-B) As described in Fig 5, PBMCs isolated from donor #3 were infected with GFP-EBV (MOI ~10) in the absence (DMSO control) or in the presence of 0.1 mM carbonic anhydrase inhibitor U-104 for 28 days. (A) At the indicated time points post-infected cells were photographed using a Fluorescent Cell Imager. Scale bars, 100 μm. (B) A fraction of cells at the indicated time points were harvested and subjected to qPCR for CA9 transcript analyses. The relative changes in transcripts (log10) of the selected genes using the 2-ΔΔCt method are represented as bar diagrams in comparison to uninfected PBMC-control using B2M as housekeeping gene. Average values +/- SEM are plotted of two independent experiments performed in similar settings. *, **, *** = p-value < 0.01, 0.005 and 0.001 respectively. (C) Heat map representation of differential gene expression pattern of the indicated viral and CA genes (CA1-CA14) of RNA-Seq data (GSE125974 and DRA011328) of B-cells infected with EBV for the indicated time points. Upregulated: red; downregulated: blue. (TIF) [file ppat.1011998.s006.TIF]

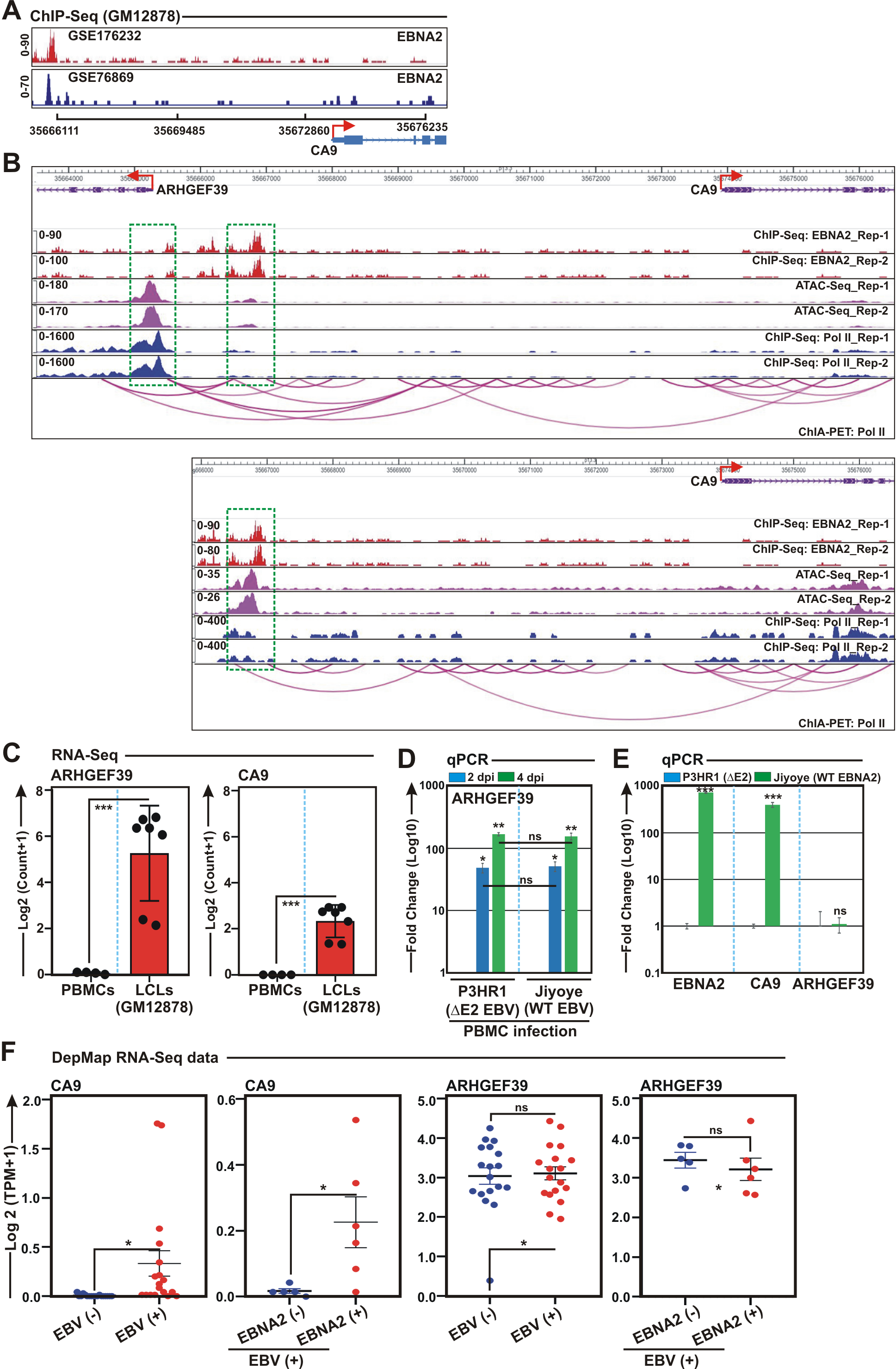

Supplement: S7 Fig — (A) Reanalyses of two ChIP-seq data (GSE176232 and GSE76869) for EBNA2 in GM12878 LCL were reanalysed and displayed using IGV software for CA9 gene locus. (B) Alignment of EBNA2 and RNA-Pol II ChIP-Seq data with ATAC-Seq and ChIA-PET for RNA-Pol II in GM12878 LCL on ARHGEF39 and CA9 genes loci. (C) Reanalyses of RNA-Seq data in PBMCs and GM12878 LCLs for CA9 and ARHGEF39 expressions. (D) PBMCs infected (MOI: ~10) with EBNA2 deleted and wild-type EBV generated from P3HR1 and Jiyoye cells, respectively for the indicated time points were subjected to qPCR analyses for ARHGEF39 expressions. (E) ~10 million P3HR1 and Jiyoye cells were harvested and subjected to qPCR analyses for the indicated viral and cell gene expressions. (D-E) The relative changes in transcripts (log10) of the selected genes using the 2-ΔΔCt method are represented as bar diagrams in comparison to control samples using B2M as housekeeping gene. Average values +/- SEM are plotted of two independent experiments performed in similar settings. *, **, *** = p-value < 0.01, 0.005 and 0.001 respectively. (F) Dot plot analyses of CA9 and ARHGEF39 expressions in response to EBV infection and EBNA2 expressions of RNA-Seq data among EBV positive and negative B-cell lymphoma cell lines in DepMap portal (https://depmap.org/portal/). (TIF) [file ppat.1011998.s007.TIF]

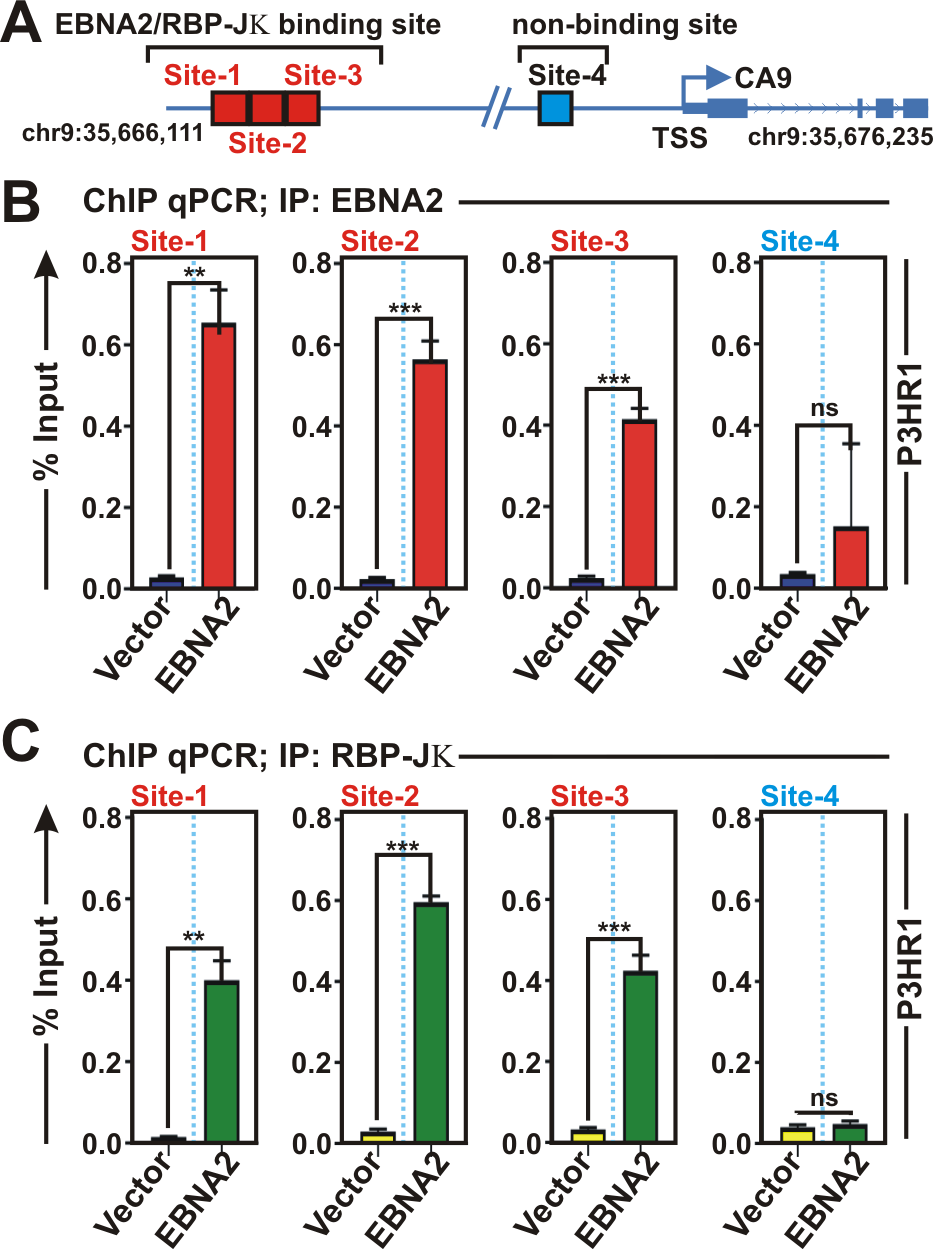

Supplement: S8 Fig — (A) Cartoon representation of genomic location for three RBP-Jκ binding and non-binding sites on the CA9 promoter region for ChIP-qPCR primer designing. Transcription start site (TSS) is indicated by red arrow. (B-C) ~20 million P3HR1 cells were transiently transfected with control vector or EBNA2 expression construct (pSG5-EBAN2) by electroporation. 48 h post-transfection cells were subjected to ChIP-qPCR analyses using EBNA2 and RBP-Jκ specific antibodies as described in the “Materials and Methods” section. Site 1, 2 and 3 are the binding region and site 4 is non-binding region for EBNA2 and RBP-Jκ as shown in (A). ChIP-qPCR primers were designed by NCBI primer BLAST application. Two independent experiments were carried out in similar settings and results represent as an average value for each genomic segment. *, **, *** = p-value < 0.01, 0.005 and 0.001 respectively. (TIF) [file ppat.1011998.s008.TIF]

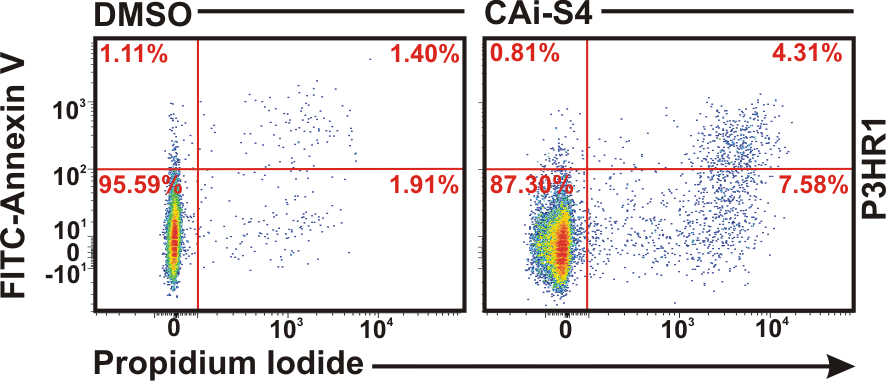

Supplement: S9 Fig — Representative images of flow cytometry analyses showing cell apoptosis in P3HR1 cells after 0.1 mM S4 treatment for 24 h. FITC labelled annexin V binding and PI staining were detected using a BD cell analyzer. Results were analysed using Floreada.io, an online based tool for flow cytometry data analyses. (TIF) [file ppat.1011998.s009.TIF]

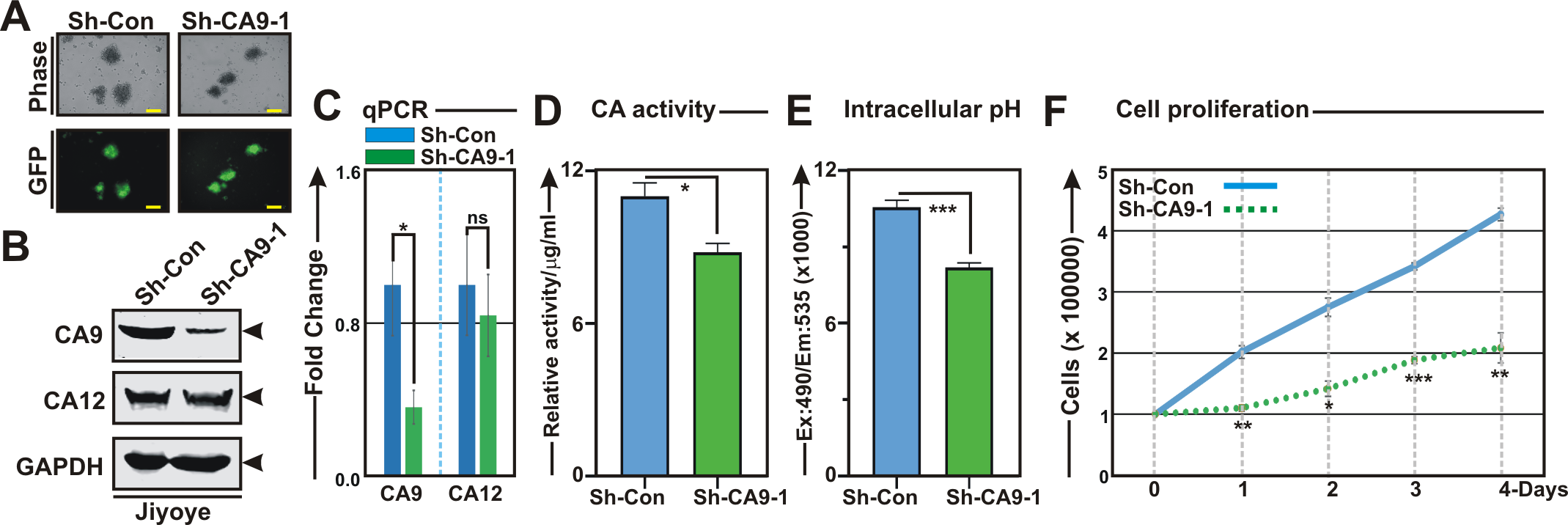

Supplement: S10 Fig — (A-F) Jiyoye cells stably transduced with lentiviruses expressing either sh-control or sh-RNA (1) against CA9 gene were (A) photographed using a using a Fluorescent Cell Imager and subjected to (B) western blot, (C) qPCR, (D) carbonic anhydrase activity, (E) intracellular pH and (F) cell proliferation analyses as similar to Fig 8O–8T. Error bars represent standard deviations of duplicate assays of two independent experiments. *, **, *** = p-value < 0.01, 0.005 and 0.001 respectively. ns: non-significant. (TIF) [file ppat.1011998.s010.TIF]

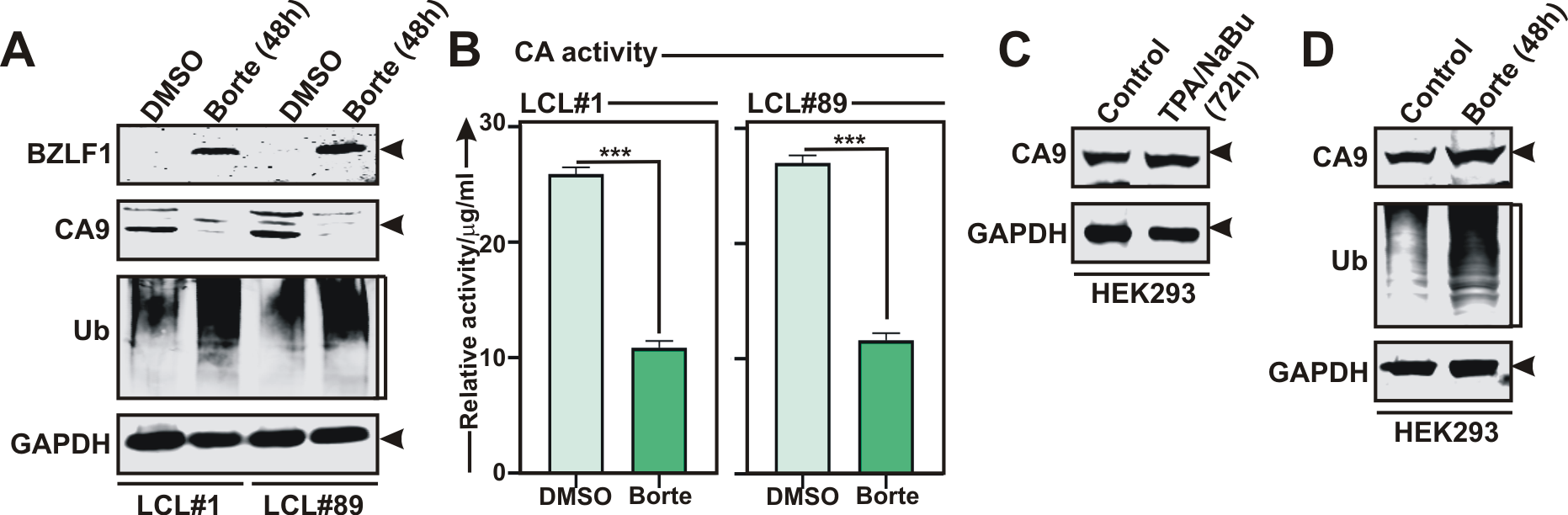

Supplement: S11 Fig — (A-C) LCLs (LCL#1 and LCL#89) either left untreated or treated with 0.5 μM bortezomib for 24 h were subjected to (A) western blot and (B) CA activity analyses. (A) For western blot analyses, GAPDH blot was performed as loading control. Representative gel pictures are shown of at least two independent experiments. (B) Carbonic anhydrase activity was measured using kit as per manufacturer’s instructions. Error bars represent standard deviations of duplicate assays of two independent experiments. *, **, *** = p-value < 0.01, 0.005 and 0.001 respectively. (TIF) [file ppat.1011998.s011.TIF]

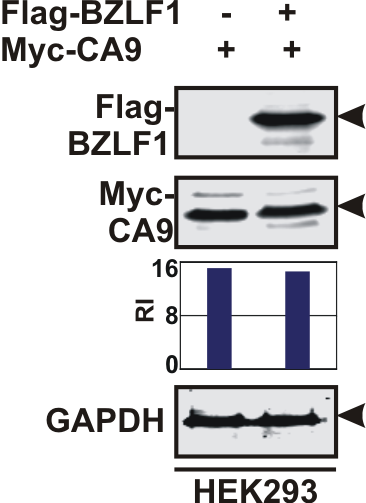

Supplement: S12 Fig — ~10 x 106 HEK293 cells were transiently transfected with myc-tagged CA9 with or without flag-tagged BZLF1 expression plasmids. 36 h post-transfection cells were harvested and subjected to western blot analyses with the indicated antibodies. GAPDH blot was performed as loading control. The relative intensities (RI) of protein bands shown as bar diagrams were quantified using the software provided by Odyssey CLx Imaging System. Representative gel pictures are shown of at least two independent experiments. (TIF) [file ppat.1011998.s012.TIF]

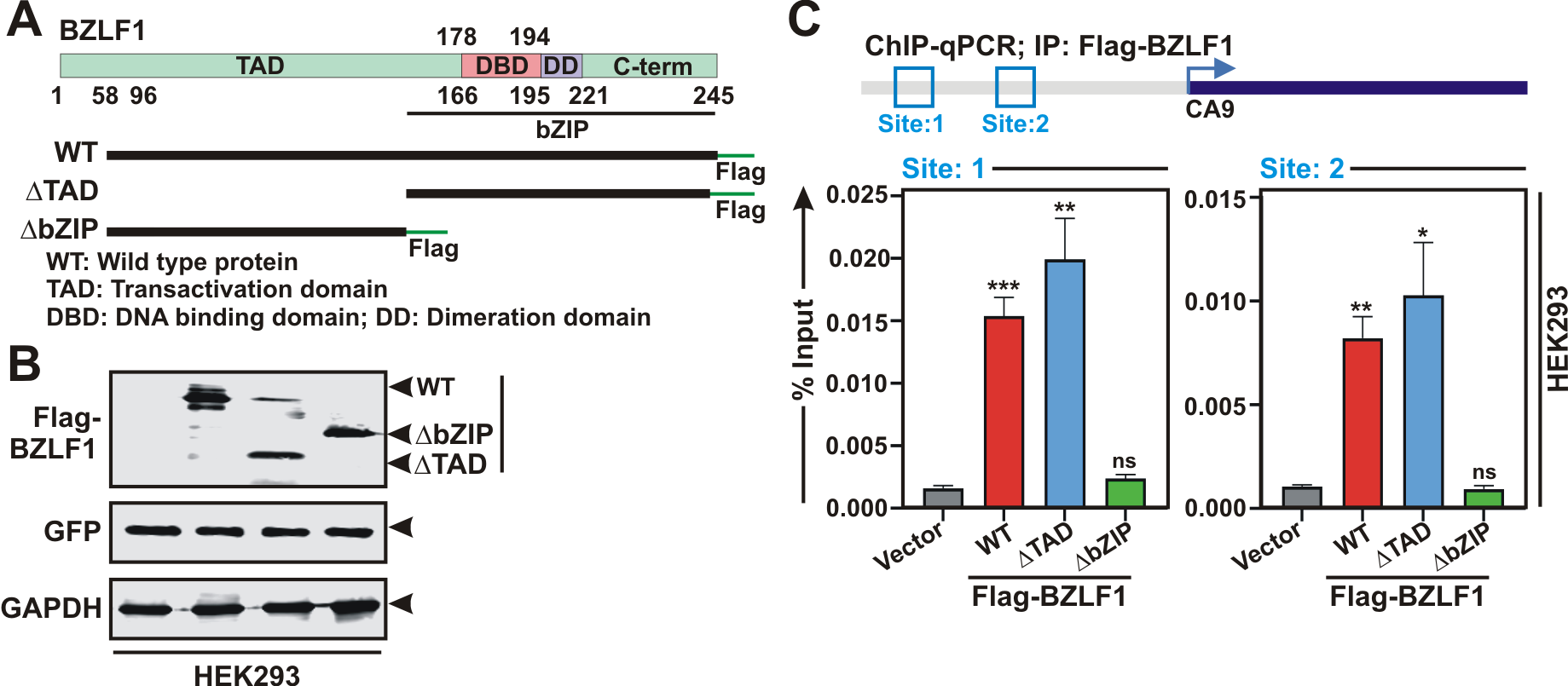

Supplement: S13 Fig — (A) Schematic representation of known structural domains of BZLF1 protein used for cloning in pA3F expression vector.(B-C) ~10 million HEK293 cells were transiently transfected with vector control or expression constructs for wild-type (WT), ΔTAD and ΔbZIP BZLF1 proteins by electroporation. 36 h post-transfection cells were harvested and subjected for either (B) western blot or (C) ChIP-qPCR analyses. (B) For western blot analyses, cells were additionally transfected with GFP expression vector to monitor the transfection efficiency. Western blots were performed with the indicated antibodies, where GAPDH blot was performed as loading control. (C) ChIP-qPCR data showing recruitment of flag-tagged wild-type or ΔTAD BZLF1 proteins on the CA9 promoter region (Site 1 and site 2). ChIP-qPCR primers were designed by NCBI primer BLAST application. Two independent experiments were carried out in similar settings and results represent as an average value for each genomic segment. *, **, *** = p-value < 0.01, 0.005 and 0.001 respectively. (TIF) [file ppat.1011998.s013.TIF]

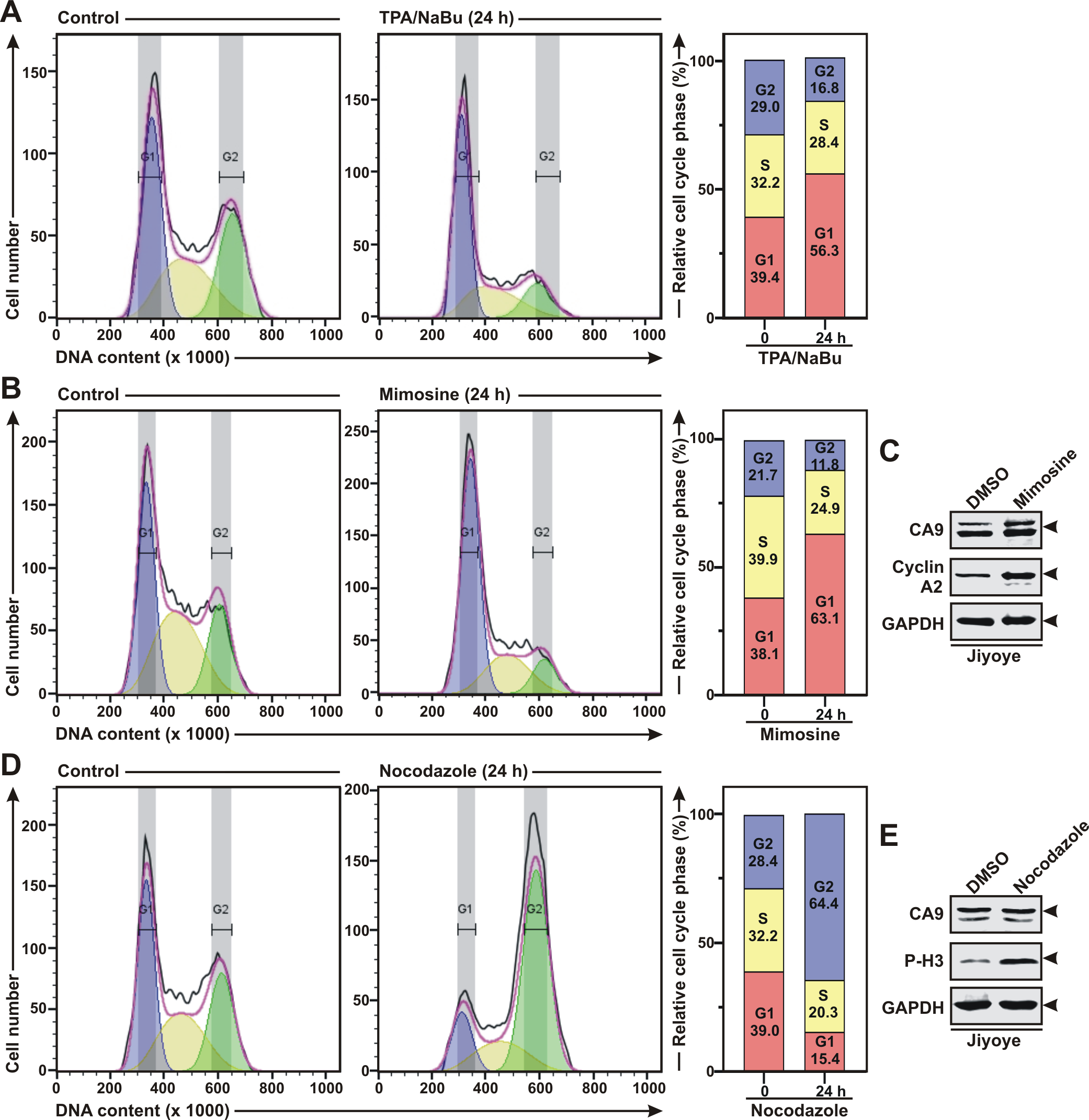

Supplement: S14 Fig — (A-E) ~10 million Jiyoye cells were treated with (A) lytic cycle inducer 20 ng/ml TPA plus 3 mM sodium butyrate (NaBu) or (B-E) cell cycle inhibitors–(B-C) 50 μM Mimosine for G0/G1 arrest or (D-E) 100 μM Nocodazole for mitotic arrest. 24 h post-treatment cells were harvested and subjected to either (A-B and D) flow cytometry or (C and E) western blot analyses. (A-B and D) Representative cell cycle distribution of propidium iodide stained Jiyoye cells using an S3e Cell Sorter. (C-E) Western blots were performed with the indicated antibodies, where GAPDH blot was performed as loading control. (TIF) [file ppat.1011998.s014.TIF]
