# Supplementary material for: Differential carbonic anhydrase activities control EBV-induced B-cell transformation and lytic cycle reactivation
Source: PLoS Pathog. 2024 Mar 26;20(3):e1011998. doi: 10.1371/journal.ppat.1011998 (PMC10997083; doi:10.1371/journal.ppat.1011998)
Supplement: S1 Table — (DOCX) [file ppat.1011998.s015.docx]

**Table S1. Ki values of carbonic anhydrase (CA) inhibitors**

| **CA inhibitors** | **Ki for CA1** | **Ki for CA2** | **Ki for CA9** | **Ki for CA12** | **References** |
| --- | --- | --- | --- | --- | --- |
| Acetazolamide | 250 nM | 130 nM | 30 nM | 5.7 nM | [1] |
| SLC-0111/U-104 | 5080 nM | 9640 nM | 45.1 nM | 4.5 nM | [2] |
| S4 | 5600 nM | 546 nM | 7 nM | 2 nM | [3] |

**References**

1. Hou Z, Lin B, Bao Y, Yan H-N, Zhang M, Chang X-W, et al. Dual-tail approach to discovery of novel carbonic anhydrase IX inhibitors by simultaneously matching the hydrophobic and hydrophilic halves of the active site. Eur J Med Chem. 2017; 132:1-10. doi:10.1016/ j.ejmech.2017.03.023

2. Lou Y, McDonald PC, Oloumi A, Chia S, Ostlund C, Ahmadi A, et al. Targeting tumor hypoxia: Suppression of breast tumor growth and metastasis by novel carbonic anhydrase IX inhibitors. Cancer Res. 2011;71: 3364-3376. doi:10.1158/0008-5472.can-10-4261

3. Gieling RG, Babur M, Mamnani L, Burrows N, Telfer BA, Carta F, et al. Antimetastatic effect of sulfamate carbonic anhydrase IX inhibitors in breast carcinoma xenografts. J Med Chem. 2012;55: 5591-5600. doi:10.1021/jm300529u
