# Supplementary material for: Differential carbonic anhydrase activities control EBV-induced B-cell transformation and lytic cycle reactivation
Source: PLoS Pathog. 2024 Mar 26;20(3):e1011998. doi: 10.1371/journal.ppat.1011998 (PMC10997083; doi:10.1371/journal.ppat.1011998)
Supplement: S2 Table — (DOCX) [file ppat.1011998.s016.docx]

**Table S2.** Previously published datasets used in this study.

| **Accession ID** | **Experiment** | **Sample** | **Cell line** | **References/URL** |
| --- | --- | --- | --- | --- |
| GSE73887 | ChIP-Seq | EBNA1 | LCL | [1] |
| GSE29498 | ChIP-Seq | EBNA2 | IB4 LCL | [2] |
| GSE176232 | ChIP-Seq | EBNA2 | GM12878 | [3] |
| GSE76869 | ChIP-Seq | EBNA2 | GM12878 | [4] |
| GSE88729 | ChIP-Seq | EBNA3A | LCL | [5] |
| GSE88729 | ChIP-Seq | EBNA3B | LCL | [5] |
| GSE88729 | ChIP-Seq | EBNA3C | LCL | [5] |
| GSE49338 | ChIP-Seq | EBNALP | IB4 LCL | [2] |
| E-MTAB-7788 | ChIP-Seq | BZLF1 | Raji | [6] |
| GSE55105 | ChIP-Seq | RelA | GM12878 | [7] |
| GSE55105 | ChIP-Seq | RelB | GM12878 | [7] |
| GSE55105 | ChIP-Seq | cRel | GM12878 | [7] |
| GSE55105 | ChIP-Seq | p50 | GM12878 | [7] |
| GSE55105 | ChIP-Seq | p52 | GM12878 | [7] |
| GSE32465 | ChIP-Seq | RUNX3 | GM12878 | [8] |
| GSE29498 | ChIP-Seq | RBPJk | IB4 LCL | [2] |
| GSE32465 | ChIP-Seq | IRF4 | GM12878 | [8] |
| GSE97661 | ChIP-Seq | BATF | GM12878 | [9] |
| GSE31477 | ChIP-Seq | EBF1 | GM12878 | [10] |
| GSE92125 | ChIP-Seq | IKZF1 | GM12878 | [10] |
| GSE53366 | ChIP-Seq | EP300 | GM12878 | [11] |
| GSE32465 | ChIP-Seq | SPI1/PU.1 | GM12878 | [8] |
| GSE127481 | ChIP-Seq | MEF2B | GM12878 | [10] |
| GSE 19551 | ChIP-Seq | RNA Pol II | GM12878 | [12] |
| GSE140203 | ATAC-Seq |  | GM12878 | [13] |
| GSE125974 | RNA-Seq |  | Resting B-cells | [14] |
| DRA011328 | RNA-Seq |  | Resting B-cells | [15] |
| SRR7251667 | RNA-Seq |  | PBMC | <https://www.ncbi.nlm.nih.gov/sra/> |
| SRR7251668 | RNA-Seq |  | PBMC |  |
| SRR7251669 | RNA-Seq |  | PBMC |  |
| SRR7251670 | RNA-Seq |  | PBMC |  |
| SRR14638511 | RNA-Seq |  | GM12878 |  |
| SRR14638512 | RNA-Seq |  | GM12878 |  |
| SRR306999 | RNA-Seq |  | GM12878 |  |
| SRR307000 | RNA-Seq |  | GM12878 |  |
| SRR307001 | RNA-Seq |  | GM12878 |  |
| SRR307002 | RNA-Seq |  | GM12878 |  |
| SRR307003 | RNA-Seq |  | GM12878 |  |
| E-MEXP-2767 | Microarray |  | BL31 | [16] |
| GSE158897 | ChIA-PET | RNA Pol II | GM12878 | <https://www.ncbi.nlm.nih.gov/geo/query/acc.cgi?acc=GSE158897> |

**References**

1. Tempera I, Leo A De, Kossenkov A V., Cesaroni M, Song H, Dawany N, et al. Identification of MEF2B, EBF1, and IL6R as Direct Gene Targets of Epstein-Barr Virus (EBV) Nuclear Antigen 1 Critical for EBV-Infected B-Lymphocyte Survival. J Virol. 2016;90: 345. doi:10.1128/jvi.02318-15

2. Zhao B, Zou J, Wang H, Johannsen E, Peng CW, Quackenbush J, et al. Epstein-Barr virus exploits intrinsic B-lymphocyte transcription programs to achieve immortal cell growth. Proc Natl Acad Sci U S A. 2011;108: 14902-14907. doi:10.1073/pnas.1108892108/

3. Hong T, Parameswaran S, Donmez OA, Miller D, Forney C, Lape M, et al. Epstein–Barr virus nuclear antigen 2 extensively rewires the human chromatin landscape at autoimmune risk loci. Genome Res. 2021;31: 2185-198. doi:10.1101/gr.264705.120

4. Gunnell A, Webb HM, Wood CD, McClellan MJ, Wichaidit B, Kempkes B, et al. RUNX super-enhancer control through the Notch pathway by Epstein-Barr virus transcription factors regulates B cell growth. Nucleic Acids Res. 2016;44: 4636-4650. doi:10.1093/nar/gkw085

5. Paschos K, Bazot Q, Ho G, Parker GA, Lees J, Barton G, et al. Core binding factor (CBF) is required for Epstein-Barr virus EBNA3 proteins to regulate target gene expression. Nucleic Acids Res. 2017;45: 2368-2383. doi:10.1093/nar/gkw1167

6. Buschle A, Mrozek-Gorska P, Cernilogar FM, Ettinger A, Pich D, Krebs S, et al. Epstein-Barr virus inactivates the transcriptome and disrupts the chromatin architecture of its host cell in the first phase of lytic reactivation. Nucleic Acids Res. 2021;49: 3217-3241. doi:10.1093/nar/gkab099

7. Zhao B, Barrera LA, Ersing I, Willox B, Schmidt SCS, Greenfeld H, et al. The NF-κB Genomic Landscape in Lymphoblastoid B Cells. Cell Rep. 2014;8: 1595-1606. doi:10.1016/j.celrep.2014.07.037

8. Gertz J, Savic D, Varley KE, Partridge EC, Safi A, Jain P, et al. Distinct properties of cell-type-specific and shared transcription factor binding sites. Mol Cell. 2013;52: 25-36. doi:10.1016/j.molcel.2013.08.037

9. Venkataraman A, Yang K, Irizarry J, Mackiewicz M, Mita P, Kuang Z, et al. A toolbox of immunoprecipitation-grade monoclonal antibodies to human transcription factors. Nat Methods. 2018;15: 330-338. doi:10.1038/nmeth.4632

10. Dunham I, Kundaje A, Aldred SF, Collins PJ, Davis CA, Doyle F, et al. An integrated encyclopedia of DNA elements in the human genome. Nature 2012 489:7414. 2012;489: 57-74. doi:10.1038/nature11247

11. Gasper WC, Marinov GK, Pauli-Behn F, Scott MT, Newberry K, DeSalvo G, et al. Fully automated high-throughput chromatin immunoprecipitation for ChIP-seq: Identifying ChIP-quality p300 monoclonal antibodies. Sci Rep. 2014;4. doi:10.1038/srep05152

12. Raha D, Wang Z, Moqtaderi Z, Wu L, Zhong G, Gerstein M, et al. Close association of RNA polymerase II and many transcription factors with Pol III genes. Proc Natl Acad Sci U S A. 2010;107: 3639-3644. doi:10.1073/pnas.0911315106

13. Ma S, Zhang B, LaFave LM, Earl AS, Chiang Z, Hu Y, et al. Chromatin Potential Identified by Shared Single-Cell Profiling of RNA and Chromatin. Cell. 2020;183: 1103-1116.e20. doi:10.1016/j.cell.2020.09.056

14. Chong W, Difei L, Luyao Z, Sizun J, Jun L, Yohei N, et al. RNA Sequencing Analyses of Gene Expression during Epstein-Barr Virus Infection of Primary B Lymphocytes. J Virol. 2019;93: 10.1128/jvi.00226-19. doi:10.1128/jvi.00226-19

15. Yanagi Y, Okuno Y, Narita Y, Masud HMA Al, Watanabe T, Sato Y, et al. RNAseq analysis identifies involvement of EBNA2 in PD-L1 induction during Epstein-Barr virus infection of primary B cells. Virology. 2021;557: 44-54. doi: 10.1016/j.virol.2021.02.004

16. White RE, Groves IJ, Turro E, Yee J, Kremmer E, Allday MJ. Extensive Co-Operation between the Epstein-Barr Virus EBNA3 Proteins in the Manipulation of Host Gene Expression and Epigenetic Chromatin Modification. PLoS One. 2010;5: e13979. doi: 10.1371/journal.pone.0013979
