# Supplementary material for: Differential carbonic anhydrase activities control EBV-induced B-cell transformation and lytic cycle reactivation
Source: PLoS Pathog. 2024 Mar 26;20(3):e1011998. doi: 10.1371/journal.ppat.1011998 (PMC10997083; doi:10.1371/journal.ppat.1011998)
Supplement: S3 Table — (DOCX) [file ppat.1011998.s017.docx]

**Table S3.** Oligo sequences used for qPCR, semi-qPCR, ChIP-qPCR, Sh-RNAs and cloning of cDNA and promoter regions.

| **SL No.** | **Gene Name** | **Reference Sequences** | **Experiments** | **Oligo Sequences** |
| --- | --- | --- | --- | --- |
|  | EBNA3A | YP_401669.1 | qPCR | Fw- 5’-GCCCTGGATGACAACATGGA-3’  Rv- 5’-CAGGTGGGCATCTTCTGCTT-3’ |
|  | EBNA3B | NC_007605.1 | qPCR | Fw- 5’-CCCTTGCGGATGCAGCCAAT-3’  Fw- 5’-GGCTGATATGGAATGTGCCC-3’ |
|  | EBNA3C | YP_401671.1 | qPCR | Fw- 5’-AAGGTGCATTTACCCCACTG-3’  Rv- 5’-AGCAGTAGCTTGGGAACACC-3’ |
|  | EBNA1 | YP_401677.1 | qPCR | Fw- 5’-CATTGAGTCGTCTCCCCTTTGGAAT-3’  Rv- 5’-TCATAACAAGGTCCTTAATCGCATC-3’ |
|  | EBNA2 | YP_401644.1 | qPCR | Fw- 5’-GAGACCAGAGCCAAACACCTCCAGT-3’  Rv- 5’-TTAGGGGTTGCCGTGTGTGAATTTC-3’ |
|  | LMP1 | YP_401722.1 | qPCR | Fw- 5’-CCCGCACCCTCAACAAGCTACCGAT-3’  Rv- 5’-TTGTCAGGACCACCTCCAGGTGCGC-3’ |
|  | BZLF1 | YP_401673.1 | qPCR | Fw- 5’- CCAGGTTGAGGTGCTTCTCCCCCGG-3’  Rv- 5’-AACCGCTCCGACTGGGTCGTGGTTT-3’ |
|  | GAPDH | NM_001289745.3 | qPCR | Fw- 5’-AATGAAGGGGTCATTGATGG-3’  Rv- 5’-AAGGTGAAGGTCGGAGTCAA-3’ |
|  | B2M | NM_004048.3 | qPCR/semi-qPCR | Fw- 5’-GAGGCTATCCAGCGTACTCCA-3’  Rv- 5’-CGGCAGGCATACTCATCTTTT-3’ |
|  | RPLPO | NM_007475.5 | qPCR/semi-qPCR | Fw- 5’-ATCTGCTTGGAGCCCACAT-3’  Rv- 5’-GCGACCTGGAAGTCCAACTA-3’ |
|  | ACTB | NM_001101.5 | qPCR | Fw- 5’- GTTGTCGACGACGAGCG -3’  Rv- 5’ GCACAGAGCCTCGCCTT -3’ |
|  | CA1 | NM_001128829.4 | qPCR | Fw- 5’- AAATGAGCATGGTTCAGAACATACA -3’  Rv- 5’ ACTTTGCAGAATTCCAGTGAGCTA -3’ |
|  | CA2 | NM_000067.3 | qPCR | Fw- 5’-GGGTACGGCAAACACAAC-3’  Rv- 5’-GGCTGTATGAGTGTCGATGTC-3’ |
|  | CA3 | NM_005181.4 | qPCR | Fw- 5’-CACACCGTGGATGGAGTCAA-3’  Rv- 5’-GTGTTATACTTCGGGTTCCAGTGA-3’ |
|  | CA4 | NM_000717.5 | qPCR | Fw- 5’-CTCTGGCTACGATAAGAAGCAAA-3’  Rv- 5’- CAGTGCAGGTGCAACTGTTT-3’ |
|  | CA5A | NM_001739.2 | qPCR | Fw- 5’- CACCTGGATCATCCAGAAGGA -3’  Rv- 5’- ACAGGAGAGTACGAAATGCAGAGA- 3’ |
|  | CA5B | NM_007220.4 | qPCR | Fw- 5’- GCCCTGCAGCCTCTATACTTGT -3’  Rv- 5’- CACGCTCTCCCAGAGTGGAT- 3’ |
|  | CA6 | NM_001215.4 | qPCR | Fw- 5’- GATGCGCCGGATGGTTT -3’  Rv- 5’- GTAATAAGTGTTTTCAGGGTAATTCTTCAC- 3’ |
|  | CA7 | NM_005182.3 | qPCR | Fw- 5’- TGGAGACAGGAGACGAGCA -3’  Rv- 5’- TCGAGGTGACACTCTCACTGA- 3’ |
|  | CA8 | NM_004056.6 | qPCR | Fw- 5’- CGGAATCGCCATCATTGCTC-3’  Rv- 5’- CGCAGCAGAGGGTCTGGTAA- 3’ |
|  | CA9 | NM_001216.3 | qPCR/semi-qPCR | Fw- 5’- GATGAGAAGGCAGCACAGAAGG -3’  Rv- 5’ - CTCTGGCTGGCTTCTCACATTC -3’ |
|  | CA12 | NM_206925.3 | qPCR/semi-qPCR | Fw- 5’-AGTGACATCCTCCAGTATGAC-3’  Rv- 5’-GTGGCACTGTAGCGAGAC-3’ |
|  | CA14 | NM_012113.3 | qPCR | Fw- 5’-CAGCCCCACGGATATGAC-3’  Rv- 5’-AGTCCACCCAGATACAGGGTA-3’ |
|  | HIF1A | NM_001530.4 | qPCR | Fw- 5’-GAACGTCGAAAAGAAAAGTCT-3’  Rv- 5’-CCTTATCAAGATGCGAACTCACA-3’ |
|  | EPAS1 | NM_001430.5 | qPCR | Fw- 5’-TTGCTCTGAAAACGAGTCCGA-3’  Rv- 5’-GGTCACCACGGCAATGAAAC-3’ |
|  | HIF3A | NM_152794.4 | qPCR | Fw- 5’-ATGCGGTCAGCAAGAGCATC-3’  Rv- 5’-AGACGATACTCTCCGACTGGG-3’ |
|  | TP53 | NM_000546.6 | qPCR | Fw- 5’-CTTCCCTGGATTGGCAGC-3’  Rv- 5’-TTTCAGGAAGTAGTTTCCATAGGT-3’ |
|  | CDKN1A | NM_000389.5 | qPCR | Fw- 5’- ACAGCAGAGGAAGACCATGTG-3’  Rv- 5’- CGTTTGGAGTGGTAGAAATCTGTC-3’ |
|  | TLR9 | NM_017442.4 | qPCR | Fw- 5’- CCCCCAGCATGGGTTTCTG-3’  Rv- 5’- GAAGTGGGGCACAGACTTCA-3’ |
|  | CD40 | NM_001250.6 | qPCR | Fw- 5’- TGATGTTGTCTGTGGTCCCC-3’  Rv- 5’- GGCTTCTTGGCCACCTTTTTG-3’ |
|  | ARHGEF39 | NM_032818.3 | qPCR | Fw-5’- AAGTGTCGATCCCTCAGCCA-3’  Rv-5’- GGATCCCCAAAAAGTACGTGG-3’ |
|  | EBNA2/RBPJκ-CA9-promoter-Site 1 | GRCh38.p14 | ChIP-qPCR | Fw- 5’- CCCAAGGAAACTGGTGGTGA -3’  Rv- 5’- CCCCAGGTGTTGTGGTCTAC -3’ |
|  | EBNA2/RBPJκ-CA9promoter_Site2 | GRCh38.p14 | ChIP-qPCR | Fw- 5’- GTAGACCACAACACCTGGGG-3’  Rv- 5’- GGCTTTGCCCTCTTCCTCTT -3’ |
|  | EBNA2/RBPJκ-CA9promoter_Site3 | GRCh38.p14 | ChIP-qPCR | Fw- 5’- GAGCATCGGTTGGGGAATCA-3’  Rv- 5’- GCCCCTCCTATCTTTGGCTG-3’ |
|  | EBNA2/RBPJκ-CA9promoter_Site4 | GRCh38.p14 | ChIP-qPCR | Fw- 5’- TCCAGGGTCAGAGTCCACAT-3’  Rv- 5’- CTTGCTTGGCCCCTGACATA-3’ |
|  | BZLF1-CA9promoter_Site1 | GRCh38.p14 | ChIP-qPCR | Fw- 5’- CCATGCTGGGATAGGCAGAG-3’  Rv- 5’- TGCCAAACCCTCAAACAGGT-3’ |
|  | BZLF1-CA9promoter_Site2 | GRCh38.p14 | ChIP-qPCR | Fw- 5’- AATTTGGCTCAGGGTCGAGG -3’  Rv- 5’- CCAGCCGCCTCTCTCTTTTA-3’ |
|  | pA3F-BZLF1 | NC_007605.1 | CDS cloning | Fw-5’-GCTGGATCC ATGATGGACCCAAACTCG-3’  Rv- 5’ATAAGAAT GCGGCCGC GAAATTTAAGAGATCCTC-3’ |
|  | pA3F-ΔTAD-BZLF1 | NC_007605.1 | CDS cloning | Fw-5’-CAGTGTGGTGGAATTCTCGCTGGAGGAATGCGAT-3’  Rv-5’- TAGACTCGAGCGGCCGCGAAATTTAAGAGATCCTCGTGTAAA-3’ |
|  | pA3F-ΔbZIP-BZLF1 | NC_007605.1 | CDS cloning | Fw-5’- CAGTGTGGTGGAATTCATGATGGACCCAAACTCGAC-3’  Rv-5’- TAGACTCGAGCGGCCGCTATTTCTAGTTCAGAATCGCATTCC-3’ |
|  | pA3M-CA9 | NM_001216.3 | CDS cloning | Fw-5’-AATAAGCTTCATGGCTCCCCTGTGCCC-3’  5’-ATAAAAGCGGCCGCGGCTCCAGTCTC -3’ |
|  | pGL3-CA9promoter-WT | GRCh38.p14 | Promoter cloning | Fw- 5’-ATAACGCGTACAGAGCAAGTCAGGCAC-3’  Rv- 5’-GTGAGATCTGGAACTTGCTTGGCC-3’ |
|  | pGL3-CA9promoter –Mutated Motif 1 | GRCh38.p14 | Promoter cloning | Fw- 5’- TTGGGAGGCTaaaaaAGGCGGATCAC -3’  Rv- 5’- AGTGCTGGGATTACAG -3’ |
|  | pGL3-CA9promoter –Mutated Motif 2 | GRCh38.p14 | Promoter cloning | Fw- 5’- GGTGGCACGTaaaaaTAATCCCAGCTAC -3’  Rv- 5’- ATGCCCGGCTAATTTTG -3’ |
|  | pGL3-CA9promoter –Mutated Motif 3 | GRCh38.p14 | Promoter cloning | Fw- 5’- TTGGGAGGCTaaaaaAGGAGAATCGC -3’  Rv- 5’- GTAGCTGGGATTACAG -3’ |
|  | pGL3-CA9promoter –Mutated Motif 4 | GRCh38.p14 | Promoter cloning | Fw- 5’- AACCCGGGGAaaaaaAGGAAACAATAAC -3’  Rv- 5’- GGGACAGAGTATACTCAG -3’ |
|  | pGL3-CA9promoter –Mutated Motif 5 | GRCh38.p14 | Promoter cloning | Fw- 5’- AGGAAACAATaaaaaAAACCTGTTTGAGGGTTTG -3’  Rv- 5’- GAGTCTCCCCGGGT -3’ |
|  | Sh-CA9-1 | NM_001216.3 | Sh-RNA Cloning | Fw-5’- tcgagtgctgttgacagtgagcgaggaagaaatcgctgaggaatagtgaagccacagatgtattcctcagcgatttcttccgtgcctactgcctcggaa-3’  Rv-5’- cgcgttccgaggcagtaggcacggaagaaatcgctgaggaatacatctgtggcttcactattcctcagcgatttcttcctcgctcactgtcaacagcac-3’ |
|  | Sh-CA9-2 | NM_001216.3 | Sh-RNA Cloning | Fw-5’- tcgagtgctgttgacagtgagcgagcaacaatggccacagtgttagtgaagccacagatgta acactgtggccattgttgcgtgcctactgcctcggaa-3’  Rv-5’- cgcgttccgaggcagtaggcacgcaacaatggccacagtgttacatctgtggcttcactaacactgtggccattgttgctcgctcactgtcaacagcac-3’ |
