# Supplementary material for: Differential carbonic anhydrase activities control EBV-induced B-cell transformation and lytic cycle reactivation
Source: PLoS Pathog. 2024 Mar 26;20(3):e1011998. doi: 10.1371/journal.ppat.1011998 (PMC10997083; doi:10.1371/journal.ppat.1011998)
Supplement: Attachment — Submitted filename: Rebuttal_Plos-Pathogens_Re-submission.pdf [file ppat.1011998.s018.pdf]

Manuscript "Differential Carbonic Anhydrase Activities Control EBV-Induced B-Cell Transformation and Lytic Cycle Reactivation" (PPATHOGENS-D-23-01533)

****

> First of all I would like to thank all three reviewers for their time and candid comments to improve our manuscript. We do appreciate the efforts placed in reviewing this manuscript and accept the positive comments and suggestions for improvement. Moreover, we have studied the reviewer's comments closely and admit that the most of the comments raised by the reviewers are important and do improve the overall strength of the manuscript. Thus we have incorporated the overall suggestions and specific comments in the revised version of the manuscript. We now think that the data in the manuscript clearly represents the strengths of the work overall and are satisfied that these changes have substantially improved the manuscript. We would like to thank the reviewers for their gracious comments as well as the editors for their time and patience in serving as editors.

Reviewer's Responses to Questions

Part I – Summary

Reviewer #1:

The authors find that Carbonic Annhydrase (CA) 9 is differentially regulated by EBV infection of naïve B-cells and upon lytic replication in lymphoblastoid cell lines. They perform RNA-seq on PBMC and demonstrate EBV infection activates CA9, and that CA9 remains elevated in established LCLs. They show that CA9 inhibitors and CA9 knock-down selectively inhibits EBV+ B-cell viability. They show that latent cycle transcription factor EBNA2 activates and binds to the promoter of CA9, while lytic activator BZLF1 represses CA9. The BZLF1 activation domain is shown to be required for the repression of CA9 during lytic replication.

>Response: We thank the reviewer for constructive comments and appropriately summarizing our findings.

Reviewer #2:

In this paper, Malik et al provide an extensive and comprehensive analysis of the impact of EBV infection and reactivation on regulating the cell membrane, cancer associated, carbonic anhydrase, CA9 expression and carbonic anhydrase activity. The manuscript contains a ton of data that delves into the regulation, the activity, and the mechanisms through which CA9 and carbonic anhydrase activity is regulated. Further, for each point, several layers of experiments are provided to provide firm assertions of findings. Overall, this is a strong and comprehensive manuscript investigating carbonic anhydrase activity through the EBV infection cycle.

>Response: We sincerely thank the reviewer for appropriately summarizing our work.

A few points to consider.

- Based on extensive literature pertaining to the role of carbonic anhydrous activity in cancer and the roles that it plays in supporting tumor growth and survival, the potential role of activating activity during infection and immortalization is an easy extension. On the other hand, it might be helpful if the authors were to provide some hypotheses for the role that suppression of CA9 during reactivation might play in supporting viral replication.

>Response: We agree with the reviewer comments. In the starting the Results section dealing with EBV lytic cycle reactivation, we wrote "While latent EBV infection in quiescent B-lymphocytes leads to B-cell activation, which ultimately develops B-cell lymphomas [1,8], EBV reactivation into lytic replication is essential to maintain the production of progeny virus and viral transmission from one host to another [54]. By partially characterized mechanisms B-cell differentiation into plasma cells provokes EBV lytic cycle reactivation [55]. Elevated CA9

expression during EBV latent infection, prompted us to further investigate CA9 expression pattern during lytic cycle reactivation." As suggested, at the end of this results section we have now added the following line in the revised manuscript

"We hypothesized that in contrast to B-cell transformation, suppression of CA9 expression and its activity might play an essential role in supporting the viral lytic cycle reactivation."

- Pertaining to down regulation of CA9 by Zta during reactivation, it would be helpful if the authors were to discuss how direct down regulation of CA9 expression by Zta would have any impact when host shut off is already programmed to shut down most cell mRNAs during reactivation through BGLF5 mediated degradation of cell mRNA transcripts.

>Response: We agree with the reviewer comments. As suggested by the reviewer, we have incorporated the following paragraph in the discussion section of the revised manuscript:

"During reactivation into lytic cycle replication, gamma-herpesviruses, including EBV, induce a global host mRNA shut-off mechanisms [92]. This is primarily accomplished by EBV encoded early protein BGLF5 mediated degradation of host transcripts along with nuclear accumulation of cytoplasmic poly(A)-binding protein [93, 94]. In addition to this canonical pathway, EBV also exerts non-canonical mechanism for blocking host gene transcriptions. For example, the immediate early lytic protein BZLF1, besides initiating viral lytic cycle replication through transcriptional activation of ~30 early lytic genes including BGLF5, can also affect host transcriptome through global restructuring of host chromatin [30]. Mechanistically, our data showed that the BZLF1 transcriptionally repressed CA9 expression through direct binding onto its promoter region. However, at this point, we cannot rule out the involvement of BGLF5 mediated post-transcriptional control of CA9 expression."

- While the authors show direct results that Zta expression can inhibit CA9 expression transcriptionally, apparently through binding to two Zta binding sites upstream from the CA9 promoter, the suppression of CA9 expression in EBV positive cells treated by TPA/Na Butyrate is much more likely to occur as a direct result of the treatment itself. It is likely that reactivation only occurs in a small percentage of cells (10%, 20%?) in which case, it would be impossible to see the observed levels of decreases in CA9 expression in the context of a background of 80%-90% of cells which are not undergoing reactivation. Relatively low levels of reactivation are partially supported by minimal induction of Zta expression in LCL#1 in figure 9E. At a minimum, the authors should make a not of this caveat.

>Response: We agree with the reviewer comments that there is a possibility that suppression of CA9 expression in EBV positive B-cells during viral lytic cycle replication initiated by the treatment of TPA/Na butyrate may depict a direct effect of the treatment itself. We have now incorporated the following statement in the corresponding results section of the revised manuscript:

"However, these results raised the possibility that CA9 expression could be affected either by direct involvement of some specific EBV lytic proteins or simply by the compound effect of these chemical inducers."

To rule out the possibility of direct engagement of treatment specific downregulation of CA9 expression, we have also induced EBV lytic cycle replication by proteasomal inhibition. Please refer to the following paragraph in the revised manuscript:

"We and others previously demonstrated that global proteasomal inhibition also can initiate EBV lytic cycle replication [11,58]. Treatment with bortezomib, an FDA approved proteasome inhibitor [59], similarly demonstrated that upon lytic cycle reactivation by proteasomal inhibition prompted downregulation of both CA9 expression and its activity in LCLs (Fig. S11A-B)."

Moreover, to nullify the direct effect, if any, of these chemical inducers on CA9 repression, HEK293 cells were incubated with either the combination of TPA and sodium butyrate or bortezomib and subjected for western blot analyses (Figs. S11C and S11D,

respectively). The results determined that neither of these chemical inducers had any direct role in alleviating CA9 expression (Figs. S11C-D). We hypothesized that in contrast to B-cell transformation, suppression of CA9 expression and its activity might play an essential role in supporting the viral lytic cycle reactivation.

- There are some concerns with the interpretation of ChIP-seq, ATAC-seq data at the CA9 promoter/enhancer region (Figure 7A). First, there are usually strong ATAC-seq signals at active promoters. So, the lack of an ATAC-seq signal at the CA9 promoter in LCL GM12878 is concerning. It would be helpful for the authors to show that CA9 is, in fact, expressed in BM12878 cells. Further, while CA9 expression in IB4 cells (where they show ChIP-seq coverage for EBNA2 and RBP-Jk) is detectable, it is quite low. Finally, the peaks (ATAC-seq and ChIP-seq (EBNA2, RBP-Jk, others)) that are discussed as a possible regulatory enhancer are very close to the promoter for the upstream gene, ARHGEF39, which is expressed at high levels in IB4 cells, for example. At a minimum, these issues should be addressed.

>Response: We again agree with the reviewer's comments. We have incorporated the following paragraph in the results section of the revised manuscript:

"However, the ATAC-Seq signal on the EBNA2 binding region was markedly lower compared to the upstream region closer to the ARHGEF39 (Rho Guanine Nucleotide Exchange Factor 39) gene locus (Fig. S7B), indicating that ARHGEF39 gene might strongly be regulated than CA9 gene in LCL background. In addition, reanalyses of RNA pol II ChIA-PET and ChIP-Seq data showed both EBNA2 binding upstream and downstream regions were linked by chromosome looping for transcriptional regulation of CA9 and ARHGEF39 (Fig. S7B). Reanalyses of RNA-Seq data of PBMCs and GM12878 LCLs indeed demonstrated that a similar phenomenon of higher ARHGEF39 expression (~ 2 fold) as compared to CA9 expression (Fig. S7C)."

In addition to this we have now incorporated several data and analyses on ARHGEF39, which overall showed that indeed EBV infection in B-lymphocytes leads to a transcriptional activation of ARHGEF39 gene (Fig. S7). However, EBNA2 has no role in this activation (Fig. S7).

<u>Reviewer #3:</u>

The study by Malik and colleagues focuses on the role of the Carbomic anhydrase CA9 during EBV infection. This work unveils distinctive expression patterns of the carbonic anhydrase isoform CA9 in latently infected naive B-lymphocytes and transformed B-lymphocytes during lytic cycle replication. CA9 plays a crucial role in maintaining cellular acid-base balance. The study demonstrates that inhibiting CA activity significantly hinders EBV-induced B-cell transformation, reduces proliferation, and impairs the colony-forming ability of transformed B-lymphocytes. This inhibition is achieved through processes like intracellular pH reduction, cell apoptosis induction, and CA9 transcript degradation. The study also highlights the significance of EBNA2-mediated CA9 transcriptional activation for the survival of latently infected B-cells and reveals that during lytic cycle reactivation, CA9 expression is suppressed by the EBV lytic cycle transactivator, BZLF1. In summary, the authors have presented compelling findings that offer valuable insights into the mechanisms exploited by EBV to facilitate B cell transformation.

>Response: We thank the reviewer for constructive comments and appropriately summarizing our findings.

Nevertheless, there is a need for more convincing evidence regarding the direct involvement of EBNA2 in the regulation of CA9 expression and the precise impact of the CA inhibitor S4 on EBV-mediated transformation.

>Response: To understand the effect of EBNA2, we isolated viruses from two EBV positive Burkitt's lymphoma lines - P3HR1 and jiyoye and subsequently infected PBMCs for 2 and 4 days (Fig. 8A). While P3HR1 contains type 2 EBV strain with naturally occurring EBNA2

deletion, Jiyoye harbours wild-type type 2 EBV strain [50,51]. Owing to EBNA2 deletion, P3HR1 virus unable to transform naïve B-lymphocytes in vitro [51] and compared to wild-type B95.8 type 1 strain, P3HR1 virus develops B-cell lymphomas less efficiently in a subset of infected cord blood-humanized mice [52,53]. qPCR analyses of PBMCs infected with either P3HR1 (ΔEBNA2) or Jiyoye (wild-type) virus demonstrated that EBNA2 expression was directly correlated with CA9 but not with CA12 expressions (Fig. 8B). However, no significant change was observed for ARHGEF39 gene, upstream of EBNA2 binding region in the CA9 promoter/enhancer region in response to either ΔEBNA2 or wild-type EBV infection of naïve B-lymphocytes (Fig. S7D), indicating that EBNA2 might only be involved in downstream CA9 regulation in that genomic region.

In addition, western blot and qPCR analyses of P3HR1 and Jiyoye cells evidently demonstrated that EBNA2 expression was directly interrelated with elevated CA9 expression, while CA12 and ARHGEF39 expression was unaltered in these two lines (Figs. 8E and S7E).

As suggested by the reviewer, a direct correlation between EBNA2 and CA9 expressions was also validated using P3HR1 null EBNA2 cell line transiently transfected with either vector control or EBNA2 expressing plasmid (Fig. 8F). The results demonstrated that EBNA2 expression stimulated CA9 expression, which was typically absent in P3HR1 cells (Fig. 8F).

ChIP-qPCR analyses also showed both EBNA2 and RBP-Jκ were significantly enriched at all three EBAN2 distal binding sites as compared to the proximal non-binding region of CA9 promoter/enhancer region in EBNA2 expressing P3HR1 cells (Fig. S8A-C). However, in contrast, no binding of both EBNA2 and RBP-Jκ were observed in vector transfected P3HR1 cells (Fig. S8A-C).

Additionally, significant CA9 expressions were also observed in both EBV positive and high EBNA2 expressing EBV positive B-cell lymphoma lines as compared to EBV negative and low EBNA2 expressing EBV positive B-cell lymphoma lines in DepMap portal (Fig. S7F). However, in contrast, no significant change was observed for ARHGEF39 expressions in both groups (Fig. S7F), indicating that EBNA2 might only be involved in transcriptional regulation for the downstream target gene CA9 expression in EBV positive B-cells.

<b>Part II – Major Issues:

<u>**Reviewer #1:**</u>

Overall, the major findings are, for the most part, technically robust. Identification of CA9 as an EBNA2 target gene that is essential for LCL formation is novel and interesting.

>Response: We thank the reviewer for encouraging comment.

The down regulation of CA9 by BZLF1 is potentially interesting, although somewhat murky mechanistically and not clearly understood functionally.

>Response: We respectfully disagree with the reviewer's comment. We performed the following experiments to mechanistically show that BZLF1 is directly involved in repressing BZLF1 expression during EBV lytic cycle replication:

- *HEK293 cells were transiently transfected with increasing concentrations of flag-tagged BZLF1 expression vector and subjected to qPCR and western blot analyses after 36 h transfection (Figs. 10A and 10B, respectively). The results demonstrated that BZLF1 expression was inversely correlated with CA9 expression in a dose dependent fashion at both transcript and protein levels (Figs. 10A and 10B, respectively).*
- *BZLF1 mediated depletion of CA9 expression in transiently transfected HEK293 cells was further corroborated with decreasing CA activity in a dose dependent manner (Fig. 10C).*

- *Previously it has been shown that BZLF1 recruits Elongin B/C-Cul2/5-SOCS-box protein ubiquitin ligase complex to facilitate p53 degradation, which appears to be essential for efficient viral propagation [62]. To determine whether BZLF1 also regulates CA9 depletion in a similar fashion, HEK293 cells transiently transfected with BZLF1 expression vector were either left untreated (DMSO control) or treated with proteasomal inhibitor MG132 for 6 h (Fig. 10D). Western blot analyses demonstrated that endogenous CA9 levels were noticeably depleted in the presence of BZLF1 expression irrespective of proteasomal inhibition (Fig. 10D), suggesting that CA9 expression might be regulated by BZLF1 mediated transcriptional repression.*
- *To further confirm BZLF1 mediated CA9 repression, HEK293 cells were transiently transfected with myc-tagged CA9 with or without flag-tagged BZLF1 expression vectors (Fig. S12). In contrast to endogenous CA9, no change in expression was observed for exogenously expressed myc-CA9 (compare Figs. 10B, 10D and S12), indicating BZLF1 mediated possible transcriptional regulation of CA9 expression.*
- *ChIP-Seq reanalyses (E-MTAB-7788) of Raji cells stably expressing BZLF1 under doxycycline responsive promoter [63] revealed several distinct BZLF1 ChIP-Seq signals in the CA9 promoter region (Fig. 10E). Model-based analysis of ChIP-Seq (MACS) tool for peak-calling with a p-value cut off set at $p<0.001$ up to ~8 Kb upstream of the TSS (transcription start site) identified two significant BZLF1 peaks at -7033 to -6430 (site 1) and -4595 to -3995 (site 2) on the CA9 promoter region (Fig. 10E).*
- *The two identified ChIP-Seq peaks in CA9 promoter were assessed for BZLF1 binding motifs using JASPAR tool and subsequently revealed four and three AP-1 motifs in the site 1 and site 2 BZLF1 binding regions, respectively (Fig. 10E).*
- *Two ChIP-qPCR primer sets flanking the site 1 and site 2 were designed and subjected for ChIP-qPCR analyses using Jiyoye cells with or without EBV lytic cycle reactivation for 72 h using TPA/sodium butyrate combination (Fig. 10F). As compared to the control, there was a significant enrichment of BZLF1 binding at both sites upon lytic cycle reactivation in Jiyoye cells (Fig. 10F).*
- *Studies suggest that chromatin accessibility by BZLF1 strictly depends on its transactivation domain (TAD), while basic leucine-zipper (bZIP) domain alone, that is, BZLF1 lacking its TAD demonstrates similar DNA binding ability [66]. In order to directly validate BZLF1 mediated CA9 transcriptional repression, we generated ΔTAD and ΔbZIP BZLF1 constructs expressing flag-tagged BZLF1 proteins lacking TAD and bZIP regions, respectively (Fig. S13A-B). ChIP-qPCR experiments using HEK293 cells transiently transfected either vector control or flag-tagged BZLF1 proteins further confirmed that only wild-type and ΔTAD BZLF1 proteins were significantly enriched at both site 1 and site 2 in the CA9 promoter region (Fig. S13C). However, no promoter enrichment was observed for ΔbZIP BZLF1 protein (Fig. S13C), indicating the importance of bZIP domain for its DNA-binding activity. Next, BZLF1 promoter region comprising of both site 1 and site 2 or mutant sites were inserted upstream of the luciferase gene in pGL3-basic vector and subsequently utilized for luciferase based promoter assay in the absence and presence of either wild-type or ΔTAD BZLF1 proteins (Fig. 10J-L). The results demonstrated that while wild-type BZLF1 transcriptionally repressed CA9 promoter activity in a dose dependent manner, ΔTAD BZLF1 did not show any transcriptional repression (Fig. 10K).*

The above-mentioned points clearly demonstrated that BZLF1 transcriptionally represses CA9 expression during EBV lytic cycle reactivation.

The role of hypoxia in regulating BZLF1 but not CA9 is confusing, and may be better left out of the manuscript.

>Response: We agree with the reviewer's comment. We have now deleted this portion in the revised manuscript.

Specific Comments:

1. According to the Methods, the initial RNA-seq was done on PBMCs, not purified B-cells, so it is difficult to conclude that the differentially regulated genes are B-cell or non-B-cell. This level of cell heterogeneity makes this experiment difficult to interpret.

>Response: We agree with the reviewer's comment. However, we have performed several following experiments using different cell line model as well as analyses using data available from the Public domain to show CA9 transcriptional activation is B-cell specific:

- *As suggested by the reviewer, we have analysed and incorporated two RNA-Seq data sets (Ref. Wang et al. J Virol. 2019;93 and Yanagi et al. Virology. 2021; 557:44-54), which utilizes purified B-lymphocytes for EBV infection in a parallel time points. As similar to our RNA-Seq data (Fig. 2A-B), reanalyses of both RNA-Seq data revealed that among all the CA isoforms, only CA9 typically expressed 2-4 DPI during EBV induced B-cell transformation (Fig. S6C).*
- *Reanalyses of RNA-Seq data of 'Genotype-Tissue Expression (GTEx; https://gtexportal.org/home/) project demonstrated that CA9 expression was significantly upregulated in EBV transformed LCLs as compared to whole blood samples (Fig. 3D).*
- *Reanalyses of microarray data of EBV infected B-cell lymphoma line BL31 (Ref. White et al. PLoS One. 2010;5: e13979) indicated significant upregulation of CA9 expression as compared to the uninfected control cells (Fig. S4).*
- *RNA-Seq analyses of both EBV positive and negative B-cell lymphoma lines in DepMap portal (https://depmap.org/portal/) also showed significant transcriptional activation of CA9 in EBV positive lines (Fig. S4).*

2. **RNAseq has been published on EBV infection of B-cells from at least 3 other groups-neither of which is referenced. The authors should also determine whether CA9 is upregulated in any of these other published studies**.

>Response: We agree with the reviewer's comments. However, we have already mentioned and analysed one RNA-Seq data (Ref. Wang et al. J Virol. 2019;93). In addition, we have incorporated analyses of another set of RNA-Seq data (Ref. Yanagi et al. Virology. 2021; 557:44-54). Please refer to the following paragraph in the results section of the revised manuscript:

"Analogous CA9 expression profiles were also observed in reanalyses of two different RNA-Seq of similar time point data sets during EBV infection of primary B-lymphocytes [42, 95] (Fig. 5F). Both our qPCR and RNA-Seq reanalyses data demonstrated that CA9 expression peaked 2-4 DPI and slowly decreased afterwards (Figs. 5E and 5F). In addition, reanalyses of both RNA-Seq data revealed that among all the CA isoforms, only CA9 typically expressed 2-4 DPI during EBV induced B-cell transformation (Fig. S6C)."

3. scRNAseq has been published on EBV infection of B-cells and tonsils and this should be cited.

>Response: We thank the reviewer for pointing this out. We have now replaced the number 7 reference with the new reference in the revised manuscript. Both papers are from Luftig MA group regarding the scRNA-Seq. However, the new reference is more appropriate. [Ref. SoRelle et al., Cell Rep. 2022; 40(9):111286]

4. Fig 8. shRNA depletion of CA9 is not as compelling as some of the pharmacological studies. CRISPR knock-down may be more efficient, or alternative targeting sequences for shRNA may also improve this knock-down.

>Response: We agree with the reviewer's comments. We have now designed a second Sh-RNA sequence and cloned accordingly in pGIPZ vector. We have performed all the experiments as similar to the previously utilized Sh-RNA directed against CA9. We have replaced the old data with the new data (Fig. 8O-T) and placed the previous data in the supplementary material (Fig. S10A-F). Please refer to the following paragraph in the revised manuscript:

"In order to determine whether EBNA2 mediated elevated CA9 expression is required for B-cell growth, CA9 expression was knocked-down using two specific sh-RNA sequences cloned in pGIPZ vector. The efficiency of CA9 knockdown by these two sh-RNAs in stably transduced Jiyoye cells was confirmed by western blot and qPCR analyses (Figs. 80-Q and S10A-C). CA9 knock-down resulted in significant reduction of both CA activity and intracellular pH in Jiyoye cells (Figs. 8R-S and S10D-E). To understand whether CA9 knockdown mediated reduction of CA activity and intracellular pH has an effect on cell growth, we conducted cell proliferation assay for 4 days (Figs. 8T and S10F). As compared to the control line, CA9 knock-down Jiyoye cells showed significant retardation of cell proliferation (Figs. 8T and S10F). Overall, the results indicated that EBNA2 mediated CA9 transcriptional activation is important for EBV induced nascent B-cell activation, B-cell proliferation and survival."

5. Fig. 10. BZLF1 is known to induce cell cycle arrest. Is the down-regulation of CA9 by BZLF1 due to cell cycle arrest? Do other cell cycle arresting agents (e.g. mimosine or hydroxyurea) also reduce CA9 expression.

>Response: We agree with the reviewer's comments. It has been suggested that in contrast to EBV latency programs, viral lytic cycle replication is associated with significant cell cycle arrest (96). EBV lytic cycle induction in both epithelial and B-cell background by various agents showed a significant G1 bias (96). Moreover, BZLF1 expression was shown to induce cell-cycle arrest at both G0/G1 and G2/M in different cell lines (97, 98). BZLF1 mediated cell cycle arrest further prompted us to examine the effect of cell-cycle arrest on CA9 expression. To this end, we first checked the cell cycle profile of EBNA2 and CA9 positive Jiyoye cells with or without the treatment of TPA/sodium butyrate for 24 h (Fig. S14A). In accordance to the previously published results (97), our data also demonstrated that EBV lytic cycle induction promotes a G0/G1 cell cycle arrest (Fig. S14A). In order to determine the effect of cell cycle arrest on CA9 expression, Jiyoye cells were either left untreated (DMSO control) or treated with 100 μM mimosine or nocodazole for 24 h (Fig. S14B-E). Mimosine, a non-protein amino acid, arrests cell cycle progression at the G1-S phase, while nocodazole is a mitotic blocker that impedes microtubule polymerization. Treatment with both mimosine and nocodazole in Jiyoye cells demonstrated characteristic cell cycle blocks at G1 and G2 phases (Figs. S14B and S14D, respectively). In addition to flow cytometry analyses, western blot data also corroborated mimosine mediated G1 phase block by accumulation of cyclin A2 protein and nocodazole mediated mitotic arrest by increased phosphor-histone 3 as compared to the untreated sample sets (Figs. S14C and S14E, respectively). However, neither mimosine nor nocodazole mediated cell cycle arrests had any repressive effect on CA9 expressions (Figs. S14C and S14E). The results therefore suggest that EBV lytic cycle induction mediated cell cycle arrest does not have any indirect role on CA9 repression.

6. Fig 10k. Interesting that BZLF1 is a potent repressor of CA9, as it is most typically thought of as a transcription activator. The authors should test whether the TAD mutant BZLF1 binds to the CA9 promoter similar to WT BZLF1 in ChIP and EMSA assay. This would determine if the TAD is required for chromatin binding or transcriptional repression more directly.

>Response: We agree with the reviewer's comments. In order to directly validate BZLF1 mediated CA9 transcriptional repression, we generated ΔTAD and ΔbZIP BZLF1 constructs expressing flag-tagged BZLF1 proteins lacking TAD and bZIP regions, respectively

(Fig. S13A-B). ChIP-qPCR experiments using HEK293 cells transiently transfected either vector control or flag-tagged BZLF1 proteins further confirmed that only wild-type and ΔTAD BZLF1 proteins were significantly enriched at both site 1 and site 2 in the CA9 promoter region (Fig. S13C). However, no promoter enrichment was observed for ΔbZIP BZLF1 protein (Fig. S13C), indicating the importance of bZIP domain for its DNA-binding activity. Next, BZLF1 promoter region comprising of both site 1 and site 2 or mutant sites were inserted upstream of the luciferase gene in pGL3-basic vector and subsequently utilized for luciferase based promoter assay in the absence and presence of either wild-type or ΔTAD BZLF1 proteins (Fig. 10J-L). The results demonstrated that while wild-type BZLF1 transcriptionally repressed CA9 promoter activity in a dose dependent manner, ΔTAD BZLF1 did not show any transcriptional repression (Fig. 10K).

7. The effects of hypoxia are difficult to interpret, particularly since hypoxia induces BLZF1 and lytic activity, but does not repress CA9, which is expected during BZLF1 lytic activation.

>Response: As suggested by the reviewer we have now deleted hypoxia related portion in the revised manuscript.

Reviewer #2:

(No Response)

Reviewer #3:

In Figure 8, it is evident that the infection with P3HR1 does not confer immortality upon primary B cells. This is due, in part, to the essential role of EBNA2, among other factors, in mediating the expression of various EBV latent transcripts. These transcripts may have a direct or indirect impact on C9A, possibly by mimicking B cell activation. This becomes more pertinent given that the authors have previously demonstrated that mitogen-induced B cell activation similarly triggers CA9 transcription (Fig. S2). Consequently, in this model, it is challenging to establish a direct correlation between EBNA2 expression and C9A expression. To substantiate their claim, the author should conduct experiments to show that depleting EBNA2 in EBV+ cell lines or introducing ectopic expression of EBNA2 has an influence on CA9 expression at both the mRNA and protein levels. This approach can also be employed to validate the binding of EBNA2 and RPB-Jk through ChIP analysis at the distal CA9 promoter enhancer region.

>Response: We agree with the reviewer's comments. As suggested by the reviewer, we have now performed the following experiments:

A direct correlation between EBNA2 and CA9 expressions was validated using P3HR1 null EBNA2 cell line transiently transfected with either vector control or EBNA2 expressing plasmid (Fig. 8F). The results demonstrated that EBNA2 expression stimulated CA9 expression, which was typically absent in P3HR1 cells (Fig. 8F). ChIP-qPCR analyses also showed both EBNA2 and RBP-Jκ were significantly enriched at all three EBAN2 distal binding sites as compared to the proximal non-binding region of CA9 promoter/enhancer region in EBNA2 expressing P3HR1 cells (Fig. S8A-C). However, in contrast, no binding of both EBNA2 and RBP-Jκ were observed in vector transfected P3HR1 cells (Fig. S8A-C).

In Figure 5, the authors demonstrated that during primary EBV infection of PBMC, the inhibition of the CA inhibitor S4 resulted in a decrease in CA activity (Fig. 5C) and a reduction in intracellular pH (Fig. 5D) over the course of the infection. Notably, qRT-PCR revealed that S4 treatment induced complete silencing of CA9 expression as early as 2 days post-infection. This observation prompts the question of whether other carbonic anhydrases play a role in the immortalization of B cells by EBV or if there are unintended off-target effects associated with S4 treatment. For instance, does the depletion of CA9 produce similar effects to those seen with S4 treatment?

>Response: We agree with the reviewer's comments. To nullify any off-target effects caused by compound S4 treatment, we performed similar EBV induced B-cell transformation experiments using another carbonic anhydrase inhibitor – U-104. As similar to compound S4, U-104 treatment also resulted in significant reduction of CA9 transcripts (Figs. 5E and S6B, revised manuscript).

Our RNA-Seq data for early phase of infection (0, 2 and 4 dpi) along with qPCR analyses for 0-28 dpi and reanalyses of two RNA-Seq data (GSE125974 and DRA011328) for similar time points (0-28 dpi) revealed that among all the CA isoforms, only CA9 expressed 2-4 dpi during EBV induced B-cell transformation (Figs. 5E-F and S6B-C). Collectively, these data suggest that EBV infection alters CA9 expression and its activity during the early infection stages to support the significant physiological shift from quiescence to rapid lymphoblastic proliferation.

Although we have not performed the B-cell transformation experiments using sh-RNAs directed against CA9 transcript, CA9 knockdown experiments in EBV positive Burkitt's lymphoma cell line Jiyoye demonstrated similar effects (retarded cell proliferation) as shown by carbonic anhydrase inhibitors.

<b>Part III – Minor Issues:

<u>Reviewer #1:</u>
(No Response)

<u>Reviewer #2:</u>
At the end of the first paragraph on page 4 of the introduction, the authors indicate that "…LCLs are treated with different agents such as immunoglobulin (IgG)…" to induce reactivation. Reactivation is not induced by IgG but instead, anti-IgG antibodies. This should be corrected.

>Response: We thank the reviewer for pointing out this inadvertent mistake. We have now corrected the sentence as suggested in the revised manuscript.

- In the first paragraph on page 16, it says, "BZLF1 promoter region comprising…". This should be "The CA9 promoter region comprising…"

>Response: We appreciate the reviewer for finding the mistake. We have now corrected the sentence as suggested in the revised manuscript.

<u>Reviewer #3:</u>
In Figure 7, the authors provided no tracks for EBNA2 binding in GM12787. However, it is worth noting that there are two datasets available for EBNA2 ChIP-seq in these cell lines, accessible through GEO under the accession numbers GSM2039170 and GSE148396. To enhance the robustness of their findings, the authors should consider integrating these datasets into Figure 7. This integration can help provide additional support for the presence of EBNA2 binding at the indicated regions. Furthermore, if discrepancies or variations in EBNA2 binding patterns are detected between these datasets and authors' observations, the authors should thoroughly discuss and analyze these differences.

>Response: We thank the reviewer for the valuable comments. As suggested by the reviewer, we have now analysed and incorporated the two EBNA2 ChIP-Seq data sets in Fig. S7A. Reanalysis of all three ChIP-Seq data revealed that EBNA2 peak was significantly enriched onto CA9 promoter/enhancer region in both GM12878 and IB4 LCLs (Figs. 7A and S7A).
